# The Difference between Growth Factor Expression after Single and Multiple Fractures: Preliminary Results in Human Fracture Healing

**DOI:** 10.1155/2015/203136

**Published:** 2015-07-13

**Authors:** Harald Binder, Stefan Eipeldauer, Markus Gregori, Leonard Höchtl-Lee, Anita Thomas, Thomas M. Tiefenboeck, Stefan Hajdu, Kambiz Sarahrudi

**Affiliations:** ^1^Department of Trauma Surgery, Medical University of Vienna, Waehringer Guertel 18-20, 1090 Vienna, Austria; ^2^Department of Internal Medicine, Gender Medicine Unit, Medical University of Vienna, Waehringer Guertel 18-20, 1090 Vienna, Austria

## Abstract

*Objectives.* Circulating levels of VEGF-A (Vascular Endothelia Growth Factor-A), TGF-*β*1 (Transforming Growth Factor-beta 1), and M-CSF (Macrophage-Colony Stimulating Factor) were found to be predictors of bone healing and therefore prognostic criteria of delayed bone healing or nonunion. The aim of this study was to evaluate a potential rise of these markers in patients with multiple fractures of long bones compared to patients with single fractured long bone. *Methods.* 92 patients were included in the study and finally after excluding all female patients 45 male patients were left for final analysis and divided into the single or multiple fracture group. TGF-*β*1, M-CSF, and VEGF-A serum levels were analysed over a time period of two weeks. *Results.* MCSF serum concentrations were higher in the group with multiple fractures as also TGF-*β*1 serum concentrations were at one and two weeks after trauma. No statistically significant difference was observed in the VEGF-A serum concentrations of both groups at either measurement point. *Conclusion.* We did observe a correlation between the quantity of the M-CSF and TGF-*β*1 expressions in serum and the number of fractured bones; surprisingly there was no statistically significant difference in the serum levels between patients with single and multiple fractures of long bones.

## 1. Introduction

Fracture healing is a unique process, which includes a complex mechanism of bone regeneration, involving several stages [1]. Numerous cytokines, angiogenic factors, proteases, and morphogens with significant roles in fracture healing have been described [2] and the bone remodelling process has been intensively investigated in the last decades. Nevertheless the vast majority of regulation mechanisms of bone healing are still unknown [3] and the knowledge and understanding of growth factors associated with fracture healing is mostly based on animal experiments or in vitro studies [4]. The search for an ideal marker for fracture healing which should ideally be on one hand quickly, easily, and noninvasively obtainable and on the other hand repetitively measurable while still being both sensitive and specific is the focus of a wide array of studies [5]. Serological markers as VEGF-A, TGF-*β*1, and M-CSF are potential factors in the complex mechanism of bone regeneration [6–9].

Evidence suggests that the local and systemic concentrations of different osteogenic growth factors increase during fracture healing [5–13]. It is also known that insufficient systemic supply of growth factors leads to loss of bone substance and to reduced differentiation of osteoblasts [14, 15]. TGF-*β*1, VEGF-A, and M-CSF are known to be pivotal for the bone healing and remodelling process [6, 7, 9, 16]. In recent studies our group demonstrated a significant increase in the concentration of these factors in fracture haematomas and in serum of patients with long bone fractures [6–8, 17]. These results indicated the importance of these cytokines for fracture healing and confirmed other clinical and experimental studies [5–13]. Circulating levels of these cytokines were found to be a predictor of delayed bone healing and nonunion in single fractures of long bones [5–13]. However to our knowledge, no data exists so far on the concentration of TGF-*β*1, VEGF-A, and M-CSF in multiple fractures of long bones. The aim of this study was to investigate possible differences in circulating TGF-*β*1, VEGF-A, and M-CSF concentrations between patients with single and multiple fractures in long bones. We hypothesized that the serum concentrations of growth factors relevant for fracture healing increase with the number of fractures and therefore analysed the correlation between the expression of these cytokines in patients with only single- and patients with multiple fractures.

## 2. Materials and Methods

This study was approved by the Ethics Committee of the Medical University of Vienna and conducted in accordance with the declaration of Helsinki. Patients gave informed, written consent to be enrolled in the study and were 18 to 90 years old. The recruitment parameters, sample collection schedule, matching process, and patient demographics as well as exclusion criteria of this study were previously published in detail [6–8]. Between April 2006 and April 2008 a consecutive series of 113 patients with meta-/diaphyseal fractures of long bones (humerus, femur, lower leg, and forearm) with surgical treatment were included. Due to the strict selection criteria 21 patients with incomplete data sets or with nonunions were excluded from further investigation, thereby reducing the number of patients to 92. These patients were then assigned to two groups, Group 1 consisting of patients who sustained a single fracture of a long bone. Groups 2 consisted of patients with multiple fractures of their long bones. Initially there were 44 female and 34 male patients with single fractures but only 2 female and 11 male patients with multiple fractures. In order to rule out gender specific differences in the expression of the cytokines and to have a homogenous study group we decided to exclude all female patients. The final analysis included 34 patients with single fractures and 11 patients with multiple fractures of long bones. Patient's demographics are shown in Tables 1 and 2.

All patients in this study showed bone healing. The diagnosis of bony consolidation or delayed union was based on exercise-induced pain and conventional X-rays or computed tomography. Delayed union was defined as failed fracture healing without radiological signs of bony consolidation after 4 months postoperatively. Nonunion was defined as the absence of complete consolidation at 6 months after surgery.

Patient's serum was collected following a standardised time schedule (1, 2, 4, 6, 8, 12, and 24 weeks after trauma). TGF-*β*1, VEGF-A, and M-CSF levels were then measured in patients' serum. All patients were followed up for at least six months after the operation. Follow-up examinations were conducted at 1, 2, 4, 6, 8, 12, and 24 weeks after trauma, including clinical and radiological examinations. Analysis of the serum samples was limited to two weeks after trauma only, due to the high number of missing samples during the later period (4–24 weeks). Consequently TGF-*β*1, VEGF-A, and M-CSF serum levels were retrospectively compared in male patients with single and multiple fractures.

### 2.1. Measurement of TGF-*β*1, M-CSF, and VEGF-A

Peripheral venous blood was obtained from each patient at one and two weeks after surgery and stored at –80°C until analysis. TGF-*β*1, M-CSF, and VEGF-A concentrations were measured by a commercially available antibody (Quantikine, RD Systems, Minneapolis, MN, USA) in enzyme-linked immunosorbent assay (ELISA). All analytical steps were performed according to the manufacturer's recommended protocol. The TGF-*β*1, M-CSF, and VEGF-A assays detect specifically the biologic active form of the protein. Concentrations are presented as mean of duplicate measurements.

### 2.2. Statistical Analysis

Comparisons between groups of continuous variables were performed by nonparametric Mann-Whitney *U* test. Spearman's correlation coefficient (male, multiple fractures of long bones, and single fractures of long bones) was used to examine the relationship between male, multiple fractures of long bones and single fractures of long bones. Statistical analyses were performed using SPSS software (Version 17.0, SPSS Inc., Chicago, IL, USA). Data are presented as mean ± SEM (standard error of the mean). The statistical significance level was set at *p* < 0.05.

## 3. Results

The average age of the patients in single fracture group was 42.9 ± 14.2 years. Patients with multiple fractures of long bones were 36.1 ± 13.5 years old (*p* = 0.125).

### 3.1. M-CSF Concentrations

**Figure 1:**
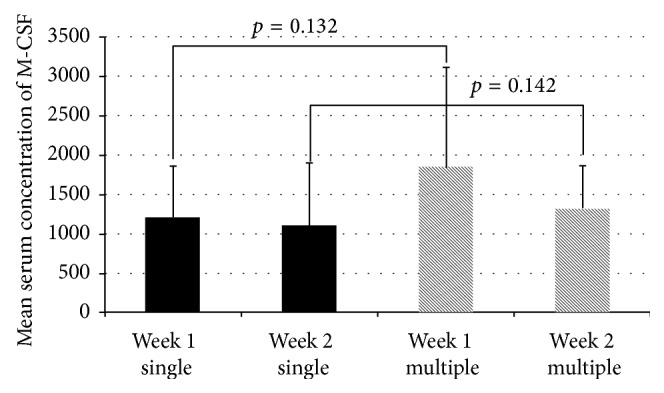
Mean M-CSF serum concentrations in patients with single and multiple fractures. W1, week 1; W2, week 2.

Mean M-CSF serum concentrations were 1212.1 ± 646.6 pg/mL at the first week and 1116.1 ± 779.3 pg/mL at the second week for the single-fracture group. For the multiple-fracture group, mean M-CSF serum concentrations were 1853.6 ± 1249.0 pg/mL at the first week and 1327.8 ± 534.5 pg/mL at the second week, respectively. Serum concentrations were higher in the group of the multiple fractures when compared to the group of single fractures. However, these differences were not statistically significant (*p* = 0.13 and *p* = 0.14). Results are revealed in Figure 1.

### 3.2. TGF-*β*1 Concentrations

**Figure 2:**
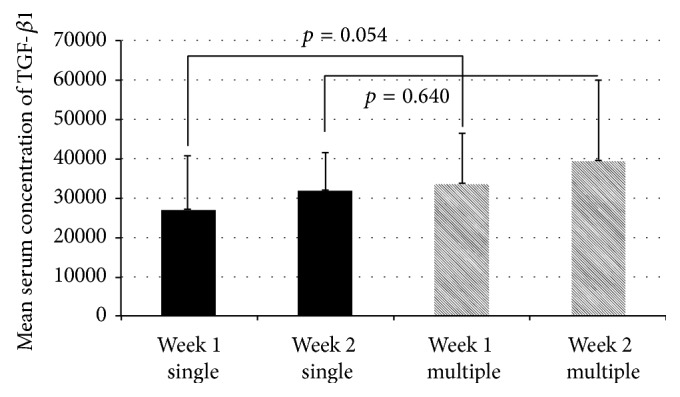
TGF-*β*1 serum concentrations in patients with single and multiple fractures.

Mean TFG-*β*1 serum concentrations measured in the single-fracture group were 27221.6 ± 13559.6 pg/mL and 32110.4 ± 9500.4 pg/mL at the first and second week after fracture, respectively. Patients with multiple fractures of long bones had higher concentrations: 33533.1 ± 13071.4 pg/mL and 39371.0 ± 20738.2 pg/mL at 1 and 2 weeks after fracture. However, the higher concentrations in the multiple-fracture group did not significantly differ from the concentrations in the single-fracture group (*p* = 0.054 for week 1 and *p* = 0.640 for week 2). Results are shown in Figure 2.

### 3.3. VEGF-A Concentrations

**Figure 3:**
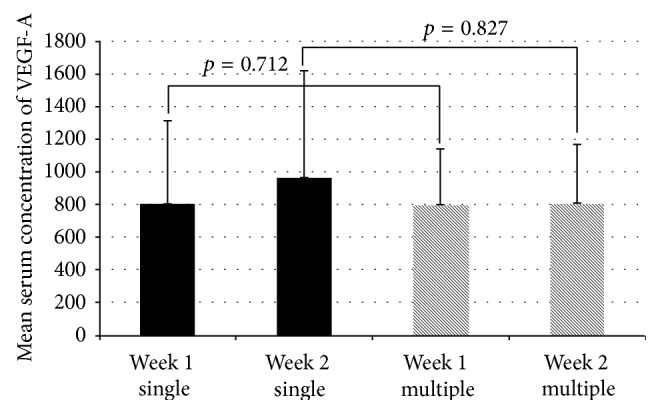
VEGF serum concentrations in patients with single and multiple fractures.

Mean VEGF-A serum concentrations were 804.1 ± 509.2 pg/mL at the first week and increased to 966.8 ± 656.7 pg/mL at the second week in patients with single fractures. Patients with multiple fractures had a nearly unchanged VEGF-A level of 793.1 ± 350.7 pg/mL and 793.3 ± 373.0 pg/mL at the first and second weeks after trauma. No statistically significant difference was observed between the concentrations of both groups at each measurement point (*p* = 0.712 for the first week and *p* = 0.827 for the second week). Results are presented in Figure 3.

## 4. Discussion

Because of the growing interest in stimulating fracture healing, detailed knowledge of the role of growth factors during the healing process is of pivotal importance. Growth factors such as BMP-7 and BMP-2 have increasingly been used for stimulation of fracture healing over the last years [18, 19]. Considering the growing importance of growth factors in clinical routine exact knowledge of the expression pattern of growth factors is essential. In previous studies we were able to demonstrate that fracture healing leads to a temporary alteration of the expression pattern as well as alteration of the quantity of the expressed growth factor in humans. These studies all showed a significant elevation of the M-CSF, TGF-*β*1, and VEGF-A concentrations in patients with bone fractures compared to healthy controls [6–8]. We could further demonstrate that initially after fracture these growth factors are produced and released within the fracture site. We presumed the growth factors present at the peripheral serum to derive from the cells at the fracture site and may induce positive feedback [6–8]. To our knowledge, based on literature search, little is known about possible alterations of the quantity of growth factor expression relating to the number of fractured bones. A recent study by Sasaki et al. showed a positive correlation between serum hyaluronan (HA) levels and the number of joints suffering from osteoarthritis (OA) [20]. It is conceivable that there is also a positive correlation between the number of fractures and the released amount of the growth factors. So we hypothesized that the quantity of the expressed growth factors relevant for fracture healing correlates with the number of the broken bones.

In the present study, we measured the level of M-CSF, TGF-*β*1, and VEGF-A in serum of male patients with fractures during the first 2 weeks after trauma. Only male patients were selected because we wanted to eliminate gender related alteration of growth factor expression as previously reported [5, 21]. Our data showed that, unlike VEGF-A, the M-CSF and TGF-*β*1 serum levels were elevated in patients with multiple fractures compared to the patients with single fractures. VEGF-A serum levels were increased in patients with single fracture compared to patients with multiple fractures. However, there was no statistically significant correlation for any of the growth factors.

These results lead themselves to the following considerations: The quantity of the M-CSF, TGF-*β*1, and VEGF-A serum levels does not change with the number of fractured bones because the growth factor expression after fracture may only be limited to the fracture zone. Possibly, the measured levels in the peripheral serum are only a small rudimentary portion of these growth factors which find their way into the serum as it was shown in different studies [6, 17, 22]. Conceivably, the serum levels of M-CSF, TGF-*β*1, and VEGF-A do change with the number of fractured bones but we were not able to show these changes because of the small number of patients narrowly selected to be included in this study. Finally, the expected alteration might occur only later during the course of fracture healing.

In summary this is the first study to evaluate the correlation between the quantity of the growth factor expression and the number of the broken bones.

*Limitations*. This study has several limitations. The first limitation is the low number of patients due to strict inclusion criteria. The second limitation is the short follow-up time. The fact that only patients with complete data were included might pose a selection bias. Moreover, we are aware of the fact that serum values might not directly reflect the ongoing local processes inside the fractured bone.

## 5. Conclusion

We did observe a trend between the quantity of the M-CSF and TGF-*β*1 expressions in serum and the number of fractured bones; however there was no statistically significant difference in the serum levels of M-CSF and TGF-*β*1 and VEGF-A between patients with single and multiple fractures of long bones. These results are valuable in revealing the characteristics of M-CSF, VEGF-A, and TGF-*β*1 serum levels as biomarkers for fracture healing. Further studies with longitudinal design would help understanding the role of these markers in human fracture healing.

## Figures and Tables

**Table 1:** Fracture localization, soft tissue damage, and treatment modalities of patients with single fractures (*n* = 34) (average age: 42.9).

Fracture	Plate/screw	IM	Ex. fix.	Gustilo 1°	Gustilo 2°	Gustilo 3°
Humerus	7	4	0	2	0	0
Radius	2	0	0	1	0	0
Ulna	1	0	0	0	0	0
Femur	0	3	1	1	0	0
Tibia	8	8	0	3	2	0

IM—intramedullary Nailing; Ex. fix.—external fixator.

**Table 2:** Demographics of patients with multiple long bone fractures.

Nr.	Age (years)	Type (ASIF-classification)	Location	Gustilo classification	Fixation
1	42	42-B2	Tibia/fibula	2	Nail/screw/plate
			Femur	0	Nail

2	63	42-A2	Tibia/fibula	0	External fixator
		11-B3	Humerus	0	Screw/plate

3	20	42-B3	Tibia/fibula and femur	3	External fixator
		32-A3		0	Nail

4	24	32-A3	Femur	0	Nail
		11-A1	Humerus		Screw

5	24	32-A3	Femur	0	Nail
		44-B2	Bimalleolar fracture	0	Screw/plate
		44-B3			External fixation

6	49	42-B3	Tibia	0	Nail
		44-B2	Bimalleolar fracture	1	Screw/plate

7	35	12-C1	Humerus	0	Screw/plate
		33-C2	Femur	2	External fixator

8	47	22-A3	Radius/ulna	0	Screw/plate
		33-A2	Femur	2	Screw/plate

9	24	42-A3	Tibia/fibula	0	Nail
			Middle phalanges	1	BD

10	40	42-A3	Tibia/fibula	0	Nail
			Metacarpales IV-V	1	Screw/plate

11	30	23-B1	Radius	0	Screw/plate
		32-A3	Femur	0	Nail
		42-A2	Tibia	0	Nail
